# D-dimer specificity and clinical context: an old unlearned story

**DOI:** 10.1186/s13054-021-03532-6

**Published:** 2021-03-10

**Authors:** Matteo Marin, Daniele Orso, Nicola Federici, Luigi Vetrugno, Tiziana Bove

**Affiliations:** 1 Department of Medicine (DAME), University of Udine, Udine, Italy; 2 Department of Anesthesia and Intensive Care Medicine, ASUFC Hospital of Udine, Via Colugna 50, 33100 Udine, Italy

D-dimer, a degradation product of activated fibrin, is considered a sensitive biomarker for thromboembolic events. Unfortunately, the D-dimer does not show as much specificity. Other conditions than venous thrombosis can also raise D-dimer level, such as pregnancy, renal failure, sepsis. An elevated D-dimer value is not sufficient to establish the diagnosis of pulmonary thromboembolism. Plasma D-dimers levels could be determined by the lysis of extra-vascular rather than intra-vascular fibrin. In the ADJUST-PE study, approximately 10% of patients with an age-adjusted D-dimer above the significant cut-off showed no angiographic evidence of pulmonary embolism [1]. In a cohort of 98 patients, Kutinsky et al. found 12 with D-dimer > 500 ng/mL who had no angiographic evidence of pulmonary embolism and 8 with D-dimer < 250 ng/mL who did have pulmonary embolism [2].

D-dimer has a negative prognostic role in the COVID-19 patient's population. Numerous studies confirm this value, although the mechanisms are not fully understood. A component responsible for high D-dimer levels could be a peculiar form of disseminated intravascular coagulation. Up to 40% of patients with COVID-19 have some form of thromboembolism (i.e., DVT or PE). However, as many as 76% of patients have an elevated D-dimer [3].

We read the review by Susen et al., which identifies D-dimer as a reliable guide for the dosage of anticoagulant therapy in COVID-19 patients [4]. Due to the previously mentioned limitations, this strategy has never been validated, even for non-COVID-19 patients. Some authors proposed a low molecular weight heparin prophylactic regimen adjusted-doses based on D-dimer levels in some specific non-COVID-19 populations. However, these populations are not comparable to the COVID-19 patients.

Furthermore, the D-dimer dose adjustment of anticoagulant prophylaxis has not been proven effective even in COVID-19 patients, although some scientific societies suggest the possibility of stratifying patients based on serum D-dimer levels. This strategy's rationale is at least controversial: even in overt disseminated intravascular coagulation (DIC), the D-dimer is unreliable since its specificity varies considerably with the cut-off value. Approximately 20% of patients with a D-dimer value greater than 2.2 μg/mL do not have DIC [5].

Finally, it should be considered that the ISTH SSC on Fibrinolysis group has identified several technical pitfalls detected in current studies on D-dimer in COVID-19 cases.

In conclusion, D-Dimer guided-anticoagulation management does not seem supported enough by evidence-based recommendations. Studies that specifically address this issue are needed before evidence-based recommendations can be made.

## Data Availability

Not applicable.
